# The Role of IgG Fc Region N-Glycosylation in the Pathomechanism of Rheumatoid Arthritis

**DOI:** 10.3390/ijms23105828

**Published:** 2022-05-23

**Authors:** Balázs Gyebrovszki, András Ács, Dániel Szabó, Felícia Auer, Soma Novozánszki, Bernadette Rojkovich, Anna Magyar, Ferenc Hudecz, Károly Vékey, László Drahos, Gabriella Sármay

**Affiliations:** 1 Department of Immunology, Eötvös Loránd University, 1117 Budapest, Hungary; balazs.tamas.gyebrovszki@ttk.elte.hu (B.G.); auerfeli@gmail.com (F.A.); novosoma@gmail.com (S.N.); 2 MS Proteomics Research Group, Research Centre for Natural Sciences, Eötvös Loránd Research Network, 1117 Budapest, Hungary; dr.andras.acs@gmail.com (A.Á.); szabo.daniel@ttk.hu (D.S.); vekey.karoly@ttk.mta.hu (K.V.); drahos.laszlo@ttk.hu (L.D.); 3 Hevesy György PhD School of Chemistry, Faculty of Science, Eötvös Loránd University, 1117 Budapest, Hungary; 4 Translational Glycomics Research Group, Research Institute of Biomolecular and Chemical Engineering, University of Pannonia, 8200 Veszprém, Hungary; 5 Central Laboratory-Microbiology Profile, Molecular Department, National Institute of Hematology and Infectious Diseases, Central Hospital of Southern Pest, 1097 Budapest, Hungary; 6 Rheumatology Department III, Polyclinic of the Hospitaller Brothers of St. John of God, 1027 Budapest, Hungary; rojkovich.b@gmail.com; 7 ELKH-ELTE Research Group of Peptide Chemistry, 1117 Budapest, Hungary; amagyar51@gmail.com (A.M.); ferenc.hudecz@ttk.elte.hu (F.H.)

**Keywords:** ACPA, galactosylation, IgG glycoforms, inflammation, rheumatoid arthritis, sialylation, TNFα

## Abstract

Anti-citrullinated protein antibodies (ACPAs) are involved in the pathogenesis of rheumatoid arthritis. N-glycosylation pattern of ACPA-IgG and healthy IgG Fc differs. The aim of this study is to determine the relative sialylation and galactosylation level of ACPAs and control IgG to assess their capability of inducing TNFα production, and furthermore, to analyze the correlations between the composition of Fc glycans and inflammatory markers in RA. We isolated IgG from sera of healthy volunteers and RA patients, and purified ACPAs on a citrulline-peptide column. Immunocomplexes (IC) were formed by adding an F(ab)_2_ fragment of anti-human IgG. U937 cells were used to monitor the binding of IC to FcγR and to trigger TNFα release determined by ELISA. To analyze glycan profiles, control IgG and ACPA-IgG were digested with trypsin and the glycosylation patterns of glycopeptides were analyzed by determining site-specific N-glycosylation using nano-UHPLC-MS/MS. We found that both sialylation and galactosylation levels of ACPA-IgG negatively correlate with inflammation-related parameters such as CRP, ESR, and RF. Functional assays show that dimerized ACPA-IgG significantly enhances TNFα release in an FcγRI-dependent manner, whereas healthy IgG does not. TNFα production inversely correlates with the relative intensities of the G0 glycoform, which lacks galactose and terminal sialic acid moieties.

## 1. Introduction

Rheumatoid arthritis (RA) is a chronic inflammatory autoimmune disease affecting the synovial membrane lining of the joints, eventually leading to irreversible joint damage and bone erosion. More than 70% of RA patients are seropositive to anti-citrullinated peptide antibodies (ACPAs) and other modified peptide antibodies (AMPAs) [1,2,3,4]. Citrullination is a posttranslational protein modification induced by the activated peptidyl arginine deiminase (PADI). ACPA is the most specific marker for RA; however, it not only has a diagnostic significance, but also, due to the pro-inflammatory properties of ACPA-IgG immune complexes, possibly contributes to the disease pathogenesis [5,6,7,8,9].

ACPA-IgG-containing immune complexes (ICs) bind to FcγRIIa on monocytes and macrophages, inducing tumor necrosis factor α (TNFα) release [5]. TNFα is the first inflammatory cytokine to appear, detected in the sera and synovial fluid of RA patients, and macrophages are the main source of TNFα in the synovium [5,10]. Three classes of FcγR are expressed on human monocytes and macrophages as well as U937 cells, a pro-monocytic, human myeloid leukemia cell line with monocyte morphology. FcγRI/CD64 is a high-affinity receptor, constitutively expressed at substantial levels. Monocytes express high levels of FcγRII/CD32, a low-affinity receptor for immunocomplexes (ICs) with two functionally distinct isoforms. FcγRIIa is an activatory receptor, triggering monocyte activation in response to receptor aggregation by immune complexes [11]. Only the activation of FcγRIIa was shown to induce TNFα production by monocytes [5]; however, the high affinity activatory FcγRI receptor is responsible for TNFα production in neutrophils [12]. FcγRIII/CD16, a receptor with moderate affinity for complexed IgG, is present only in a low level on circulating monocytes [7].

The mechanism behind the inflammatory property of ACPA is incompletely understood. Glycosylation of IgG Fc is considered a critical regulator of the antibody effector functions. Mature Fc glycoforms are complex biantennary sugar moieties at asparagine 297 of the IgG CH2 domains [13]. The core Fc glycan is composed of four N-acetylglucosamine (GlcNAc) and three mannose residues [14]. The core glycan can be modified by additional sugars, including a core fucose, bisecting GlcNAc, as well as galactose and sialic acid at one or both arms [15].

Aberrant glycosylation of IgG in inflammatory autoimmune diseases has been observed [16,17,18], as well as a significantly lower level of N-galactosylation and sialylation, whereas higher fucosylation was detected on IgG from RA patients compared to healthy controls [19,20]. Sialylation has shown an inverse correlation with disease activity. According to a recent model, glycosylation of IgG is immunologically regulated, and the lower sialylation of IgG in RA is a result of the enhanced number/function of follicular T (Tfh) cells, and, in particular, Tfh17 cells downregulating sialyltransferase β-galactoside α-2,6-sialyltransferase 1 (ST6Gal I) in autoantibody-producing B cells. Consequently, the generated IgG is deficient in terminal sialic acid residues [21,22]. There is a close correlation between systemic inflammation and ACPA-IgG1 Fc sialylation in RA and other autoimmune diseases [18]. Low levels of total serum IgG or autoantibody sialylation have been observed in patients with a variety of autoimmune diseases such as RA, antiphospholipid syndrome (APS), vasculitis, or SLE [20,23,24,25,26]. Some studies suggest that the two CH2 domains of highly sialylated IgG might be more likely to form a closed conformation, impairing the binding to FcγR [27,28,29]. However, others reveal no or a slight effect of sialylation on IgG Fc structure and an unaltered binding affinity to activating FcγR [27,30,31].

To investigate the functional differences between immune complexes formed by ACPA-IgG and control IgG from healthy volunteers, we applied U937 cells, a pro-monocytic, human myeloid leukemia cell line with monocyte morphology, as a model to study the pro-inflammatory activities of small (dimeric) IC. ACPA-IgG complexes induced significantly higher TNFα release compared to control IgG and was dependent on the binding of IC to FcγRI. Determining the site-specific N-glycosylation profile of the IgG Fc region in the same cohort, we observed an inverse correlation between TNFα production and the relative intensity of N4H3 glycan corresponding to the G0 glycoform. Analyzing the correlations with inflammation-related markers, we found a negative correlation between RF, CRP, and ESR values and the level of both galactosylation and sialylation of ACPA-IgG. We also confirmed earlier results showing that both the sialylation and galactosylation level of ACPA-IgG subclasses is significantly lower compared to IgG from healthy controls.

## 2. Results

Comparative analysis of N-glycans on IgG purified from sera of healthy volunteers and on ACPA-IgG of RA patients.

Our aim was to reveal whether aberrant N-glycosylation of IgG ACPA influences the effector function of the antibodies associated with inflammation, namely, the capacity of ACPA to induce the in vitro release of inflammatory cytokine TNFα. We collected sera from anti-cyclic citrullinated peptide (CCP)-positive antibodies in diagnosed RA patients and from healthy controls with ethical permission and purified the IgG fractions by protein G affinity chromatography. Sera of RA patients were tested for ACPA-IgG by ELISA using different citrulline-containing peptides corresponding to citrullinated epitopes in filaggrin, collagen, and a multi-epitope citrulline peptide characterized by us previously (see Table in Section 8) [32]. The ACPA reactivity and demographical and clinical data of the patients are summarized in Table 1. ACPA was affinity purified on the corresponding citrulline peptide-coated columns. The eluted and flow-through fractions were tested for purity by SDS-PAGE and re-tested for peptide specificity (data not shown).

IgG from healthy controls, ACPA-IgG, and flow-through fractions from the citrulline peptide-coated columns were digested with trypsin and the glycosylation pattern of glycopeptide fragments were analyzed by determining site-specific N-glycosylation using nano-UHPLC-MS/MS.

**Figure 1:**
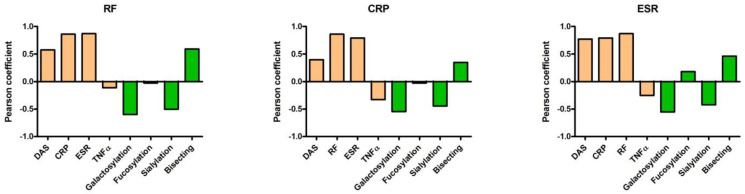
Correlations of RF, CRP, and ESR of patients with the other inflammatory markers, with ACPA-IC-induced TNFα production, and with the level of galactosylation, fucosylation, sialylation, and bisecting GlcNAc of IgG1 ACPA, based on testing 17 RA patients. Pearson coefficients are shown.

First, we analyzed the correlations between inflammation-related parameters of RA patients and the levels of the main glycoforms. Based on the analysis of 77 glycopeptides from IgG Fc N-glycan, the galactosylation level of ACPA-IgG highly negatively correlated with RF titers and showed a moderately negative correlation with CRP and ESR. The sialylation level showed a moderately negative correlation with all three markers, whereas the level of fucosylation, which mainly affects the binding of IgG to FcγRIII [33,34] showed no correlation. Interestingly, the ACPA-IC-induced in vitro TNFα production did not correlate with the clinical inflammatory markers, whereas the level of bisecting GlcNAc moderately correlated with RF and ESR. We also observed a highly positive correlation between RF, ESR, and CRP (Figure 1).

We compared the relative intensity values of galactosylation and sialylation of ACPA-IgG1, IgG2, and IgG3 subclasses from RA patients and IgG1, IgG2, and IgG3 from healthy controls. N-glycopeptides from the different isotypes of IgG were differentiated based on their amino acid sequences. The discrimination of intact IgG3 and IgG4 N-glycopeptides with high similarity in sequence is not possible with the method used [35]; thus, these were measured together but are labeled “IgG3” in the text.

**Figure 2:**
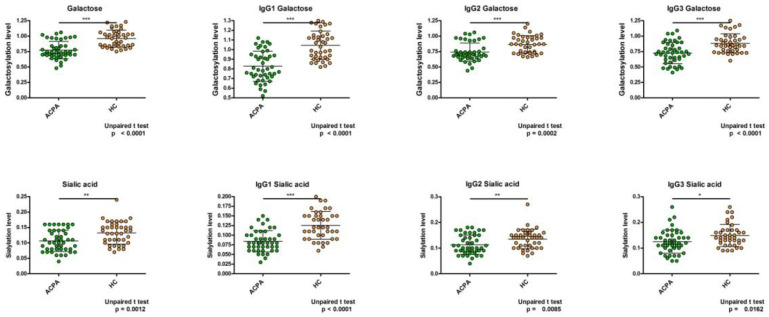
The relative intensity values of galactosylation and sialylation are significantly lower in ACPA-IgG compared to control IgG subclasses. The first column shows the results obtained with the total IgG. *n*: ACPA = 44, HC = 40. * *p* < 0.05, ** *p* < 0.01, *** *p* < 0.001.

Both galactosylation and sialylation levels of all IgG subclasses were significantly lower in ACPA compared to healthy samples, confirming earlier results where the analysis of glycans was carried out with a different method: linear ion-trap electrospray ionization mass spectrometry (LTQ-ESI-MS) (Figure 2). The highest differences were detected in the IgG1 samples.

**Figure 3:**
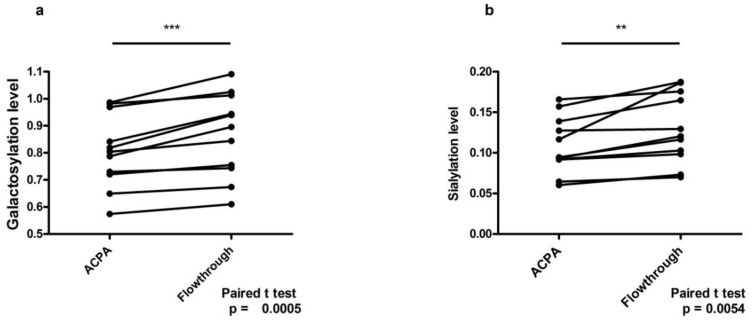
Comparison of galactosylation (**a**) and sialylation (**b**) levels of ACPA-IgG and non-ACPA IgG (flow-through fractions) from the same patients (*n* = 11). ** *p* < 0.01, *** *p* < 0.001.

To exclude individual differences from the analysis, we also compared ACPA-IgG and non-ACPA IgG (the flowthrough fractions from the citrulline peptide columns) from the same patient. The level of galactosylation and sialylation was significantly lower in ACPA compared to non-ACPA fractions, with the latter falling in the range of healthy control IgG (Figure 3).

## 3. Dimeric Immune Complexes of ACPA-IgG and Control IgG Bind to FcγRI on U937 Cells

**Figure 4:**
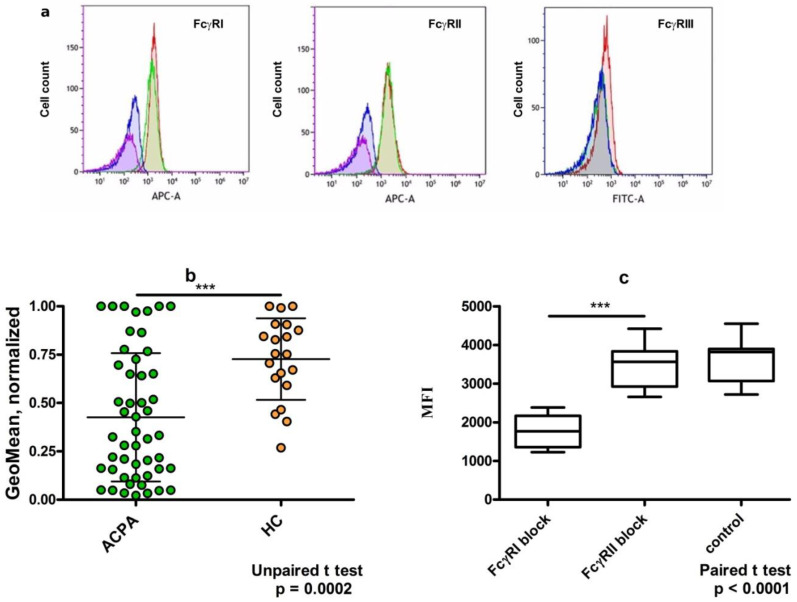
Binding of ACPA-IgG and control IgG from healthy volunteers to FcγRI on PMA-stimulated U937 cells. (**a**) Expression of FcγRI, FcγRII, and FcγRIII on U937 cells. FcγRI and FcγRII were detected by indirect immunofluorescence using eFluor 660-conjugated secondary antibodies; FcγRIII was detected by direct staining with FITC-labeled FcγRIII-specific antibody. Unstained cells: purple; secondary antibodies or isotype control: blue; specific antibodies: green and red lines for unstimulated and PMA-stimulated U937 cells. (**b**) Binding of ACPA and healthy control IgG containing immune complexes to PMA-stimulated U937 cells. Immune complexes were prepared by dimerizing IgG with Alexa Fluor 647-conjugated F(ab)2 fragments of anti-human IgG Fab. (**c**) Preventing ACPA-IC binding to U937 cells by anti-FcγRI but not by anti-FcγRII-blocking antibodies. Control: untreated cells. *** *p* < 0.001.

We chose U937 cells as a model cell line to study the binding and functional activity of various IgG immune complexes. The immune complexes were formed by adding F(ab)’_2_ fragments of anti-human IgG Fab labeled with Alexa 647 to purified ACPA or control IgG (10 µg/mL). The stoichiometry of the components allows the formation of dimeric IgG complexes, which has in vivo relevance [12]. The expression of FcγR on PMA-stimulated U937 cells was checked by monitoring the binding of specific antibodies against FcγRI, FcγRII, and FcγRIII by flow cytometry. As expected, FcγRI and FcγRII were readily detected but FcγRIII was only negligibly expressed on the cell line (Figure 4a). Next, we compared the binding capacity of dimeric immune complexes formed by ACPA-IgG and healthy IgG and found that both complexes bound to the U937 cells, but ACPA complexes exhibited a decreased mean fluorescent intensity compared to healthy IgG (Figure 4b). To test whether type I or type II FcγR were responsible for the binding, we applied blocking antibodies. After pre-treating the cells with FcγRI- or FcγRII-specific blocking antibodies, we observed that only anti-FcγRI could inhibit the binding, indicating that the dimeric immune complexes can occupy primarily FcγRI on the cell surface (Figure 4c).

## 4. Dimeric ACPA Immune Complexes Induce Significantly Higher TNFα Release from U937 Cells Compared to Complexes Containing IgG from Healthy Individuals

**Figure 5:**
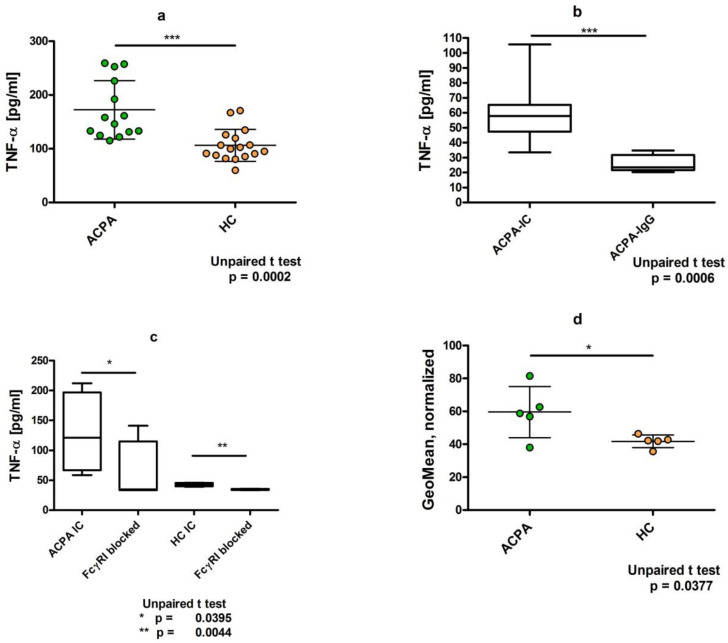
Immune complexes of ACPA induce significantly higher TNFα production compared to immune complexes of healthy control IgG. (**a**) PMA stimulated U937 cells were treated with pre-prepared immune complexes for 24 h, the supernatants were harvested, and TNFα was measured from the supernatants by ELISA (*n* = 14 (ACPA), *n* = 17 (HC)). (**b**) Immune complexes from ACPA but not the monomeric ACPA-IgG can stimulate TNFα release from U937 cells (*n* = 8). (**c**) Anti-FcγRI-blocking antibodies inhibit ACPA IC-induced TNFα release from U937 cells (*n* = 4). (**d**) Intracellular detection of TNFα synthesized by U937 cells in response to ACPA-IC. TNFα was detected in fixed and permeabilized cells by PE-conjugated anti-TNFα antibodies (*n* = 5). * *p* < 0.05, ** *p* < 0.01, *** *p* < 0.001.

Pro-inflammatory activity of immune complexes is mediated through their binding to the activating FcγR and the induction of TNFα production. Therefore, we compared dimeric immune complexes by measuring TNFα from the supernatants of U937 cells pre-treated with IC-containing ACPA IgG or control IgG complexes for 24 h. Pre-formed complexes were prepared for 30 min at 37 °C and then added to the cells. ACPA-IgG complexes triggered a significantly higher TNFα secretion compared to healthy IgG, indicating that despite the lower binding to the cells, ACPA-IgG has higher pro-inflammatory activities (Figure 5a). On the other hand, when only ACPA IgG was added to the cells without the crosslinking with the F(ab)2 fragment of anti-IgG Fab antibodies, we could not observe any difference in TNFα production compared to the untreated control cells (Figure 5b). To assess FcγR dependency of TNFα production, we pre-treated the cells with FcγRI-blocking antibody, which, similar to the IC binding, significantly reduced IC-induced TNFα release from U937 cells (Figure 5c).

Finally, to control whether ACPA IC indeed triggers TNFα synthesis, the IC-treated cells were additionally stimulated with PMA and ionomycin in the presence of Golgi stop, Brefeldin A for 5 h and TNFα was detected in the fixed and permeabilized cells by intracellular staining. We observed a significantly higher intensity of staining with the PE-conjugated anti-TNFα antibody in ACPA-IC-treated compared to control IgG-treated U937 cells, indicating that ACPA IC binding to FcγRI indeed induces TNFα synthesis (Figure 5d).

## 5. TNFα Production Induced by ACPA-Containing Immune Complexes Shows a Negative Correlation with the Relative Percentage of N4H3 Glycan of IgG Fc

**Figure 6:**
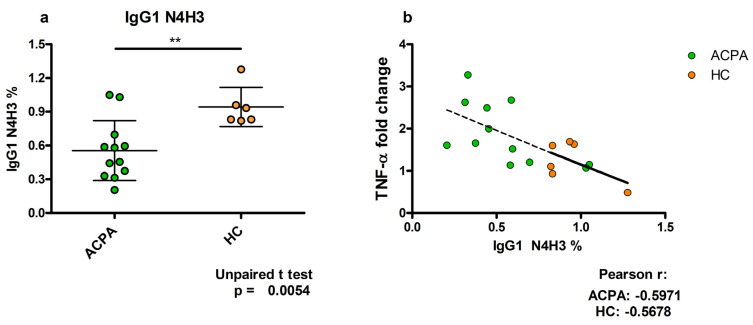
Association of the relative percentages of the N4H3 glycan in ACPA IgG1 with its TNFα-inducing capacity. (**a**) ACPA IgG shows a significantly lower N4H3 glycan level compared to control IgG. (**b**) The relative percentages of N4H3 inversely correlate with the induced TNFα production. ** *p* < 0.01.

TNFα plays important functions in the pathogenesis of RA; TNFα antagonists are widely applied in therapy and have shown remarkable efficacy in various immune-mediated inflammatory diseases, including RA. ACPA-containing IC was shown to induce TNF-α production by macrophages [5]. Our aim here was to reveal whether the TNFα-inducing capacity of ACPA IC associates with the altered glycoforms of ACPA-IgG Fc. We identified a total of 77 glycopeptides in ACPA IgG Fc; of these only the N4H3 representing the agalactosylated IgG with the biantennary oligosaccharide terminating in GlcNAc showed a high negative correlation with the TNFα-inducing capacity of ACPA-IC (Figure 6).

## 6. Discussion

ACPA might contribute to RA pathogenesis through a variety of mechanisms. It binds directly to citrullinated self-antigens on the surface of cells or interact with different Fc receptors, thereby modulating the function of many cell types [9]. N-glycosylation of IgG Fc at Asn 297 highly influences the effector functions. The glycosylation pattern of ACPA-IgG differs from that of healthy controls; the lower level of ACPA-IgG sialylation and galactosylation is associated with higher inflammatory properties [23,25,36]. Sialic acid binds to the biantennary structure of the glycan terminating in one or two galactoses; thus, agalactosylated IgG Fc (IgG G0 form) has no sialic acid and has high proinflammatory activity [15,16]. Autoantibodies with low sialic acid and/or no galactose form circulating immune complexes that can activate inflammatory cells, eventually resulting in the development of autoimmune diseases. However, the underlying mechanism of how hyposialylated and hypogalactosylated IgG might induce inflammation is not purely understood. The inflammatory potential of ACPA IC was first demonstrated by Clavel et al., show demonstrated that the insolubilized immune complexes induce TNFα release via engagement of the activating FcγRIIa on macrophages [5]. Other studies have shown that soluble dimerized IgG ACPA complexes preferentially bind to FcγRI on activated neutrophils, stimulating potential effector mechanisms by which ACPA could contribute to RA pathogenesis [12]. Additionally, it was found that ACPA and RF working in conjunction elicit a synergistic effect via IC formation enhancing inflammation [10,37].

The aim of this study was to reveal whether the structure of N-glycan on ACPA-IgG Fc correlates with its pro-inflammatory capacity in vivo and in vitro. To study the in vitro effect, we used the PMA-activated U937 cell line, which expresses FcγRI and FcγRII and practically no FcγRIII, and monitored TNFα release induced by ACPA-containing and IgG-containing IC. In vivo inflammation was followed by monitoring inflammation-associated factors such as RF, CRP, and ESR in the same cohort of patients. The N-glycan profile of ACPA-IgG and healthy IgG was assessed by comparing the relative percentages of galactose, sialic acid, and fucose in the glycopeptides we identified.

Comparing the level of galactosylation and sialylation with the clinical inflammatory markers, we found that both showed an inverse correlation with RF, CRP, and ESR (Figure 1). This is in concert with the previously described high pro-inflammatory capacity of hyposialylated, hypogalactosylated ACPA IgG [15,16,19,23].

The level of fucosylation did not correlate with clinical inflammatory markers, and a low positive correlation was found with the level of bisecting GlcNac. IgG1 Fc glycans lacking a core fucose residue (afucosylated Fc glycans) have higher affinity for FcγRIIIa (and FcγRIIIb) [33]. FcγRIIIa mediates antibody-mediated cellular cytotoxicity (ADCC) and plays a role in virus infections [34], but apparently does not influence inflammation. The effects of bisecting GlcNAc on IgG function are not well understood. Some studies indicate a role for bisection in the modulation of FcγRIIIa-mediated activities; however, data on this are not consistent [38,39].

Interestingly, the in vitro TNFα-inducing capacity of ACPA-IgG complexes did not correlate with the clinical inflammatory markers of patients, indicating that although TNFα plays important functions in the pathogenesis of RA, it is only one element of a complex network regulating inflammatory processes. Although the systemic effect of ACPA-IC-induced TNFα production cannot be seen, it still might have a local effect on the inflamed synovium. Furthermore, TNFα can also be produced by T cells independently of FcγRI, which might make a major contribution to the development of chronic inflammation in RA patients.

Analyzing the glycosylation level of ACPA-IgG, in agreement with previous results [20], we found that all subclasses of ACPA IgG had a significantly lower level of galactosylation and sialylation compared to control IgG subclasses (Figure 2). Thus, aberrant glycosylation may influence the effector functions mediated by all IgG subclasses.

We compared galactosylation and sialylation levels of affinity-purified ACPA-IgG and the flow-through fractions from the peptide column used for the isolation containing the non-ACPA IgG of the same patient. Both galactosylation and sialylation levels of non-ACPA-IgG was significantly higher compared to ACPA-IgG, indicating that the IgG glycosylation level is reduced only in the pathogenic antibody-secreting cells (Figure 3). This is in concert with a recent model suggesting that the lower sialylation of IgG in RA is a consequence of the enhanced number/function of follicular T (Tfh) cells that negatively regulate sialyltransferase β-galactoside α-2,6-sialyltransferase 1 (ST6Gal I) in autoantibody-producing B cells. [21,22].

Immune complex-binding assays showed that ACPA-IgG dimers bound to FcγRI on U937 cells with lower intensity, providing a lower MFI compared to control IgG (Figure 4b). Sialylation of IgG may modulate its function by binding to sialic acid-binding immunoglobulin-like lectins (Siglecs), such as Siglec-9 and Siglec-3 (CD33) [40]. Siglec-9 is expressed at a very low level, but CD33 is highly expressed on U937 cells and macrophages [41]. Thus, sialylated IgG from healthy volunteers might have simultaneously occupied both FcγRI and CD33, resulting in a higher binding avidity to the cells. On the other hand, Siglec-3 is an immune-modulatory receptor with an immunomodulatory tyrosine-based inhibitory motif (ITIM) within its intracellular domain. After being phosphorylated in FcγR-activated cells, ITIM recruits SHP-1 and SHP-2 phosphatase down-regulating activation signals. Such a mechanism might contribute to the anti-inflammatory effect of highly sialylated IgG.

In earlier studies it was shown that ACPA IC stimulated TNFα release by binding to FcγRIIa, the activating receptor on macrophages [5]. In this study we found that despite the lower binding to U937 cells, most importantly, ACPA-IgG dimers but not monomeric ACPA triggered a significantly higher TNFα secretion compared to control IgG dimers (Figure 5a,b). We detected TNFα both in the supernatants of cells and as an intracellularly synthesized protein. Blocking antibodies to FcγRII did not affect either the binding of ACPA dimers to U937 cells or the enhanced TNFα production, whereas FcγRI-blocking antibodies partially prevented both effects (Figure 5c). We suppose that the different composition and size of immune complexes might explain the difference between earlier findings and the present results. Insolubilized large immune complexes can bind to the lower-affinity FcγRIIa, but in our experiments, however, dimerized IgG preferentially interacted with the high-affinity FcγRI and induced TNFα secretion (Figure 5). Although in vivo FcγRI is probably occupied by serum IgG, immune complexes might compete with IgG binding to the de novo expressed FcγRI on activated cells, as suggested previously regarding activated neutrophils [12]; thus, the supposed mechanism might have in vivo relevance.

Fc glycan modifications directly affect the ability of IgG to interact with effector cells and dysregulation of the modifications can lead to loss of immune tolerance and autoimmunity, and infectious diseases [36]. Galactosylation of the Fc glycan is mediated by β-1,4-galactosyltransferase 1 (B4GALT1) and terminal sialylation is catalyzed by α-2,6-sialyltransferase 1 (ST6GAL1), which adds sialic acid to the α-1,3 arm of the biantennary glycan [36], giving rise to the G1, G2, and G2S glycoforms with increasing anti-inflammatory activity [15]. Downregulation of ST6GAL1 in inflammatory autoimmune diseases may lead to the production of hyposialylated IgG by autoantibody-producing cells [42]. A direct role for galactosylated Fc glycans in the modulation of immune functions has not been defined. However, it was shown that IgG galactosylation associates with disease activity in pregnant women with RA [43]. Furthermore, galactosylation, independent of sialylation, is associated with improvement of RA during pregnancy [44].

We examined whether the capacity of ACPA-IgG complexes eliciting TNFα secretion depends on the IgG Fc glycan. Remarkably, we could not detect an association of TNFα production with the level of sialylation; however, in the set of 77 glycovariants analyzed, we identified one glycopeptide with the structure of N4H3, which showed a highly significant negative correlation with the TNFα-inducing capacity. This glycan did not contain galactose or sialic acid, derived from the G0 form of IgG that possesses the highest proinflammatory activity. A high level of the G0 form of IgG has been observed in several autoimmune diseases [25]. Thus, association of IgG G0 with the FcγRI-dependent TNFα production might contribute to the pathogenic effect of the autoantibodies in inflammatory autoimmune diseases in a sialylation-independent way.

## 7. Conclusions

We found that both sialylation and galactosylation levels of ACPA-IgG negatively correlate with inflammation-related clinical parameters in RA. Functional assays showed that dimerized ACPA-IgG significantly enhances TNFα release in a FcγRI-dependent manner, whereas healthy IgG does not. Finally, ACPA-IgG-induced TNFα production inversely correlates with the relative intensities of the G0 form of the IgG Fc glycan. We suggest that the association of agalactosylated IgG G0 with the FcγRI-dependent TNFα production might contribute to the pathomechanism of inflammatory autoimmune disorders such as RA.

## 8. Materials and Methods

### 8.1. Blood Samples

Blood samples were collected from RA patients diagnosed according to the revised classification criteria of the American College of Rheumatology/European League Against Rheumatism (ACR/EULAR) [45]. Blood samples were taken after the patients signed a written consent. The study was conducted in accordance with the Declaration of Helsinki, and the protocol was approved by the Ethics Committee of the Ministry of Human Resources, Deputy State Secretary for National Chief Medical Officer, Department of Health Administration (21390-6-2017/EÜIG). Anti-CCP antibodies were obtained from positive female patients (*n* = 17, average age = 58 years). Age- and sex-matched control sera were obtained from healthy volunteers at the university (*n* = 20, average age = 52 years) who had not been vaccinated in the last three months and had no inflammatory or autoimmune diseases. Blood samples were collected in Vacuette 9 mL Z Serum clot activator tubes (Greiner Bio-one).

### 8.2. Serum Preparation

Fresh samples of blood were incubated at room temperature for an hour and then centrifuged at 800× *g* for 10 min. Then, the red blood cell-free serum was aspirated from the supernatant and stored in protein LoBind (Eppendorf) tubes at −20 °C until further use.

### 8.3. Determination of Anti-Citrullinated Protein Antibodies (ACPA) in Sera by ELISA

The ACPA content of the acquired serum samples was detected with an indirect ELISA as previously described [32,46,47]. Shortly, ELISA plates were pre-coated with 5 μg/mL neutravidine overnight at 4 °C to enhance peptide binding, and then were coated with selected citrulline or arginine-containing biotinylated peptides (see Table 2 for amino acid sequences) at 1 μg/mL concentration for an hour at 37 °C. Blocking was carried out with 2% BSA, 150 mM NaCl in PBS for 30 min at 37 °C. After rinsing, sera samples were added in a 1:100 dilution, and the plates were incubated overnight at 4 °C. After several washing steps, rabbit anti-human IgG(H+L)-HRPO detection antibody (Southern Biotech) was added in a 1:15,000 dilution and incubated at 37 °C for an hour. Development was carried out using TMB (Sigma) and the reaction was stopped using 2N H_2_SO_4_. The results were evaluated via photometry and OD indexes (OD on citrulline-containing peptide/OD on arginine-containing peptide) were determined. When the OD index >1.5 the sample was considered positive for ACPA and recommended for affinity purification on the corresponding citrulline peptide column.

### 8.4. Isolation of IgG

IgG was isolated from serum samples on Protein G columns (Sigma) according to the manufacturer’s instructions. One mL of sample was measured onto the column with 1 mL PBS and left flowing through until the first visually detectable yellow drop appeared at the output valve. At this point the column was closed and incubated with the sample for 30 min at room temperature. Columns were then washed with 10 mL PBS and then IgG was eluted with 100 mM glycine, pH 2.5, into 1.5 mL tubes containing neutralization buffer. The eluted fractions were tested for protein content (Nanodrop ND-1000, Absorbance at 280 nm). Protein concentration was adjusted to 1 mg/mL and then dialyzed against PBS. The dialyzed samples were collected and stored at −20 °C until further use.

### 8.5. ACPA Isolation

ACPA were affinity purified from IgG fractions of RA patients on citrulline-containing peptides (provided by Anna Magyar and Katalin Uray at ELTE Peptide Chemistry Research Group) immobilized on NHS (n-hydroxi-succinimide) columns (Hi Trap^®^, GE Healthcare, Uppsala, Sweden) as previously described [32]. The concentration of the eluted fractions was determined and adjusted to the 0.5–1 mg/mL range. The samples were then dialyzed against 50 mM NH_4_HCO_3_ buffer 2 h at room temperature and then with a fresh batch of buffer overnight at 4 °C. Dialyzed fractions were checked again for IgG concentration and specificity, and then stored at −80 °C until further use.

### 8.6. Preparation of Immune Complexes

Artificial immune complexes (IC) were generated from 10 µg/mL ACPA-IgG or IgG from healthy controls by crosslinking two IgG molecules to form a dimer by adding Alexa Fluor 647-conjugated F(ab_2_)’ fragment of anti-human IgG Fab (16 µg/mL) (Jackson ImmunoResearch) at the adequate stoichiometry [12] and incubated at 37 °C for an hour.

### 8.7. Immune Complex Binding Assay

U937 cells (ATCC, CRL-1593.2™) were used to study the binding and pro-inflammatory function of immune complexes. The monocyte-like human histiocytic lymphoma cell line U937 can be induced by phorbol 12-myristate 13-acetate (PMA) to undergo differentiation into a macrophage-like phenotype. Prior to adding IC, U937 cells were stimulated with 50 ng/mL PMA for 24 h. After stimulation, the cells were washed in sterile PBS and the IC were added to the cells and incubated for 30 min on ice in the dark, then washed with 1% FCS-PBS. The binding was measured via flow cytometry on a Beckman Coulter Cytoflex S flow cytometry system. The fluorescent signal was detected in the APC channel and the geometric means were calculated for each artificial immune complex. Giving the crosslinking F(ab2)’ alone served as a negative control, since it is not to adhere to U937 cells.

To identify the FcγR responsible IC binding, we used FcγR-blocking antibodies. For FcγRI: anti-CD64 monoclonal antibody (10.1), eBioscience™; for FcγRII: anti-CD32 monoclonal antibody (AT10), Absolute antibody Ltd. The same antibodies were used to detect FcγRI and FcγRII on the cell surface. The binding was detected by F(ab’)_2_ anti-mouse IgG-eFluor660 (Invitrogen). To detect FcγRIII, FITC-labeled anti-CD16 (Abcam) was used, and the respective signals were detected via flow cytometry.

### 8.8. Intracellular Staining to Monitor Inflammatory Cytokine TNFα Production

TNFα production was measured 24 h after IC addition to PMA-stimulated U97 cells. Five h prior to preparation a mixture of PMA (50 ng/mL), ionomycin (1 ug/mL), and brefeldin-a (×1000 dilution according to manufacturer protocol), also known as a PIB cocktail, was added to the cell cultures. Then, the cells were centrifuged at 300 rcf for 5 min at 4 °C, and supernatant was collected and stored at −20 °C until further use. BD Fix-Perm was added, and the cells were then incubated for 20 min on ice. An equal amount of BD Perm-Wash was added, and the cells were then centrifuged at 300 rcf for 5 min at 4 °C and the supernatant was discarded. The washing step was repeated with double the amount of BD Perm-Wash. Then, anti-TNFα antibody PE conjugate was added to the cells in BD Perm-Wash and incubated on ice for 30 min, washed out three times with BD Perm-Wash, and measured via flow cytometry. In all cases, the geometric mean of the fluorescent signal was calculated and compared.

### 8.9. TNFα Detection in the Supernatant of IC-Treated U937 Cells

As for the TNFα release assay, the previously PMA-stimulated U937 cells were incubated with the preformed IC made of 10 µg/mL IgG or ACPA and 16 µg/mL crosslinking F(ab2)’ at 37 °C in a 5% CO2 atmosphere for 24 h, and then the cells were centrifuged at 300 rcf for 10 min and the supernatants were collected and stored at −20 °C until further use. The supernatants were tested for inflammatory cytokine release with a BioLegend ELISA Max™ anti-human TNFα ELISA kit according to the manufacturer’s protocol.

### 8.10. Glycosylation Analysis

Samples collected from ACPA and total serum IgG isolation procedures were reduced, alkylated, and digested in solution with trypsin and Trypsin/Lys-C Mix at 37 °C as previously described [48]. A nano LC-MS(MS) system was used for peptide separation and glycopeptide analysis. The chromatographic separation was carried out on an Ultimate 3000 nanoRSLC system (Dionex, Sunnyvale, CA, USA). Samples were desalted using an Acclaim PepMap100 C-18 trap column (Thermo Scientific, Sunnyvale, CA, USA) and peptide separation was achieved with an Acquity UPLC M-Class Peptide BEH C18 column (Waters, Milford, MA, USA). A Maxis II ETD Q-TOF (Bruker Daltonics, Bremen, Germany) equipped with a CaptiveSpray nanoBooster ion source was used for the mass spectrometry measurements. Glycopeptides were identified by scanning the MS spectra over the mass range of m/z 150−3000 at 2 Hz. CID analysis was performed on triply charged precursor ions at 0.5 Hz for low-abundance ones (>2500 cts/s) and 4 Hz for abundant precursors (>25,000 cts/s). Glycopeptide quantification was based on MS experiments performed over the mass range of m/z 300−3000 at 1 Hz.

### 8.11. Statistical Analysis

Recalibrated data were generated by Compass DataAnalysis 4.3 (Bruker Daltonics, Bremen, Germany). Glycopeptides were identified by Byonic v3.8.13 (Protein Metrics, Cupertino, CA, USA) and confirmed by manual evaluation. Glycopeptide intensities were quantified by assessing the AUC (area under the curve) of triply charged precursor ion intensities with a software developed in house called GlycoPattern 4.7_b30 [49]. All values were normalized to the total IgG1 Fc glycopeptide abundance. The degree of galactosylation (G), sialylation (S), and fucosylation (F) and the frequency of bisecting N-acetylglucosamine (GlcNAc, N) residues were calculated by summing up the values of glycopeptides containing the respective sugar residues. The value of glycopeptides with two residues of the same kind (i.e., two galactoses) were multiplied by two. The following formulas were used: galactosylation: N4H4 + N4H4F1 + N4H4S1 + N4H4S1F1 + N3H4F1 + N3H4S1F1 + N5H4 + N5H4F1 + N5H4S1F1 + 2·(N4H5 + N4H5F1 + N5H5F1 + N4H5S1 + N4H5S1F1 + N5H5S1F1 + N4H5S2F1), sialylation: N4H4S1 + N4H5S1 + N4H4S1F1 + N4H5S1F1 + N5H5S1F1 + N3H4S1F1 + N5H4S1F1 + 2·(N4H5S2F1), fucosylation: N4H3F1 + N4H4F1 + N4H5F1 + N5H5F1 + N4H4S1F1 + N4H5S1F1 + N5H5S1F1 + N4H5S2F1 + N5H3F1 + N3H4F1 + N3H4S1F1 + N5H4F1 + N5H4S1F1, bisecting GlcNAc: N5H5F1 + N5H5S1F1 + N5H3F1 + N5H3 + N5H4 + N5H4F1 + N5H4S1F1.

### 8.12. Further Statistical Analysis

Statistical analysis was carried out with the use of GraphPad Prism 5 and MS Excel software.

Data from FcγR-blocking experiments and ACPA/flow-through experiments were tested with a two-tailed paired *t* test. Data from MS, IC binding, and TNFα release experiments were tested with a two-tailed unpaired t test to compare the difference between RA and healthy control groups.

## Figures and Tables

**Table 1:** Citrulline peptide specificity, demographic, and clinical data of the investigated RA patients. Citrulline peptide reactivity is expressed as the OD index, which is the optical density of the sample on citrulline-containing peptide divided by the optical density of the corresponding arginine-containing peptide.

Demographic and Clinical Parameters of Patients						Citrulline Peptide Specificities		
Age (Years)	DAS	RF IU/mL	CCP IU/mL	CRP mg/mL	ESR mm/h	Multi-Pitope	Collagen	Filaggrin
						5.86	7.8	8.53
73	2.10	93.7	871	12.6	12	4.21	6.12	2.03
47	5.07	55	3200	0.67	20	1.45	1.27	4.22
72	7.20	1695	2424	34	95	2.48	1.48	3.24
65	3.70	53	3200	3		8.81	2.4	3.74
67	6.90	105		8.6	48	1.85	1.15	3.3
72	3.80	392	906	13.1	17	1.96	1.97	3.62
65	3.30	16.3	510	1.82	7	0.6	4.58	4.41
54	4.00	74	3090	5.9	14	4.42	5.87	4.29
65	3.70	53		2.2	12	1.98	5.87	4.29
67	5.20	63	653	0.3	38	1.05	1.39	1.91
57	2.10	34	3200	1.5	3	8.53	4.21	10.18
66	3.80		70	8.4	19	0.99	1.13	1.24
53	4.40	346	50	2.5	23	1.85	0.97	0.65
73	5.20	974	700	30	89	2.84	20.67	16.1
44	3.10	51.8	281	16.88	14	2.32	4.07	1.41
66	5.10	262	1633	10.5	24	4.38	5.67	3.19

**Table 2:** Sequences of citrulline-containing peptide.

Synthetic Citrulline-Containing Peptides	Sequences (X Stands for Citrulline) *
Filaggrin19 (306–326)	Ac-SHQESTXGXSXGRSGRSGSK-NH2
Collagen (359–369)	Ac-AXGLTGXPGDA-NH2
Multi-Epitope	H-Ttds **-AXAXGSGSGXGXG-NH2

* In ELISAs the native forms of the peptides-containing arginine was also used. ** Ttds: Ttds, linker 1,13-diamino-4,7,10-trioxatridecan-succinamic acid.

## Abbreviations

ACPA	anti-citrullinated protein antibodies
CCP	cyclic citrullinated peptides
CRP	C-reactive protein
DAS	disease activity score
ESR	erythrocyte sedimentation rate
FcγR	IgG Fc receptor
GlcNAc	N-acetyl glucosamine
IC	immune complexes
PMA	phorbol myristate acetate
RA	rheumatoid arthritis
RF	rheuma factor
SDS-PAGE	sodium dodecyl sulphate polyacrylamide gel electrophoresis
TNFα	tumor necrosis factor α
Tfh	follicular helper T cells
